# An open-source anthropomorphic robot hand system: HRI hand

**DOI:** 10.1016/j.ohx.2020.e00100

**Published:** 2020-02-24

**Authors:** Hyeonjun Park, Donghan Kim

**Affiliations:** Dept. of Electrical Engineering, Kyung Hee University, Republic of Korea

**Keywords:** Anthropomorphic robot hand, Multi-finger end-effector, Modular robot finger, Four-bar linkage mechanism, Under-actuated mechanism

## Abstract

We present an open-source anthropomorphic robot hand system called HRI hand. Our robot hand system was developed with a focus on the end-effector role of the collaborative robot manipulator. HRI hand is a research platform that can be built at a lower price (approximately $500, using only 3D printing) than commercial end-effectors. Moreover, it was designed as a two four-bar linkage for the under-actuated mechanism and provides pre-shaping motion similar to the human hand prior to touching an object. A URDF, python node, and rviz package is also provided to support the Robot Operating System (ROS). All hardware CAD design files and software source codes have been released and can be easily assembled and modified. The system proposed in this paper is developed with a five-finger structure, but each finger is modularized, so it can be developed with end-effectors of various shapes depending on the shape of the palm.

## Graphical abstract

**Figure:**
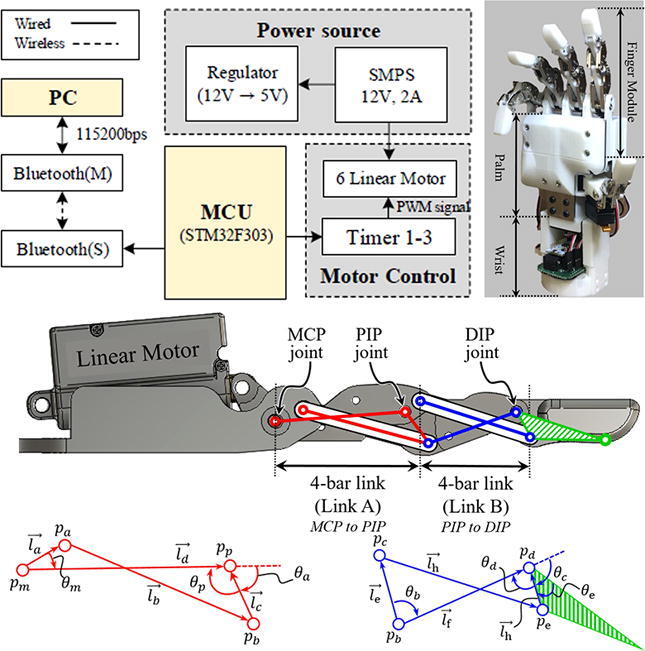

Specifications table

**Table:** 

Hardware name	HRI hand (Human-Robot Interaction LAB hand)
Subject area	Robotics engineering Electrical engineering
Hardware type	Anthropomorphic robot hand multi-finger end-effector
Open source license	MIT license
Cost of hardware	$500 for if only 3D printing is used, $400 for SUS304 processing
Source file repository	https://osf.io/sfpb2/ DOI https://doi.org//10.17605/OSF.IO/SFPB2

## 1 Hardware in context

**Fig. 1:**
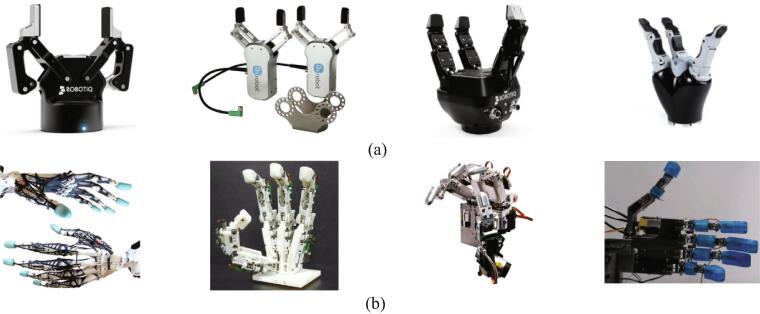
Two types of fingered end-effectors: (a) gripper type [9], [21], (b) anthropomorphic type [10], [11], [12], [13].

Collaborative robots are designed to perform tasks in collaboration with workers in industrial sectors [1]. The role of collaborative robots is growing in these new manufacturing environments. In particular, end-effectors are being researched to grip and assemble various objects beyond the simple pick-and-place operation of parts [2], [3], [4], [5], [6], [7], [8]. The robotic hand of a collaborative robot, that is, an end-effector, has various forms, but a fingered end-effector is the focus of this paper and is classified into two types: the gripper type [9] and the anthropomorphic (multi-finger) type [10]. The gripper type is the simplest form of an end-effector and is most commonly used in the industrial field (Fig. 1a). It is usually a two-finger gripper or three-finger gripper, and it picks up objects with opening and closing motions [2], [3], [4]. However, the gripper type can conduct only simple tasks, such as picking up an object, and it has limitations in cases where a machine needs to be operated or when tasks requiring precise operation need to be performed. The anthropomorphic type mimics the human hand and has the appearance of a multi-finger configuration (Fig. 1b). To collaborate with humans, collaborative robots should be able to handle various tools in the same space as humans. Therefore, the anthropomorphic type is more capable for broader applications than the simple gripper type. However, for this anthropomorphic type, it is necessary to secure a large number of degrees of freedom (DoF), which requires a corresponding number of actuators, complex mechanisms, and control algorithms [10], [11], [12], [13], [14], [15]. Several research studies are robot end-effector open-source projects. Dollar et al. [16] proposed an adaptive and compliant grasper (two-fingered gripper), which is constructed using polymer-based shape deposition manufacturing (SDM). This gripper is actuated by a single DC motor without the aid of any sensory feedback. Ma et al. [17] developed a modular 3D-printed under-actuated end-effector (four-fingered gripper). The hand is designed with a hybrid pulley/whiffletree differential mechanism and flexure joints, which are made of low-cost materials and 3D-printed parts (less than $500). Tlegenov et al. [18] proposed a robotic end-effector platform for facilitating research on robotic grasping. This gripper is actuated by a single servo motor without sensory feedback and three-fingered under-actuated mechanisms. Krausz et al. [19] developed a six DoF anthropomorphic type robot hand. The hand has one DoF for each finger, with coupled MCP and PIP joints, and two DoF for the thumb: one for flexion/extension and one for rotation.

We present the hardware and software of our open-source anthropomorphic robot hand system for experiments in a collaborative robot, which we call the HRI hand. The HRI hand is a research platform that can be built at a lower price (approximately $500, using only 3D printing) than a commercial end-effector. Moreover, it is designed as a two four-bar linkage for the under-actuated mechanism and provides pre-shaping motion similar to the human hand prior to touching an object [20]. Additionally, the robot finger is modularized and researchers can use it as an end-effector with the desired shape according to the design of the palm. Each finger is actuated by one linear motor. The thumb part has an extra motor for an abduction/adduction. For controlling all fingers, the micro-controller unit (MCU) using NUCLEO-F303K8 and can receive control signals by Bluetooth wireless communication. A URDF, python node, and rviz package are also provided to support the Robot Operating System (ROS) [22].

## 2 Hardware description

**Fig. 2:**
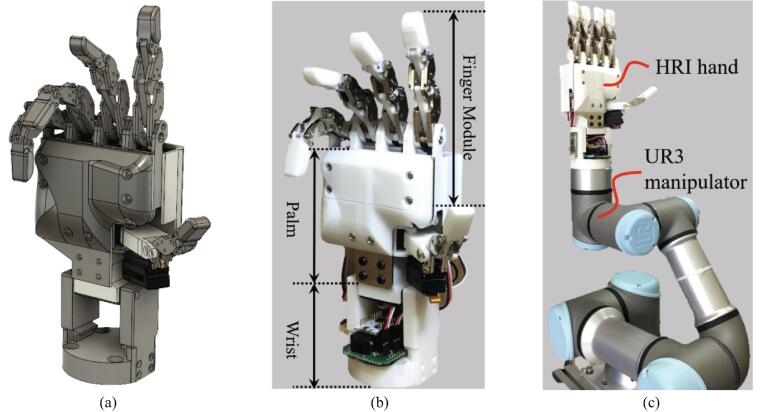
HRI hand: (a) 3D modeling, (b) proposed robot hand, and (c) HRI hand with UR3 manipulator.

The proposed robot hand is identical in the joint structures because it mimics a human hand (Fig. 2a-b). The four fingers, excluding the thumb, consist of distal interphalangeal (DIP), proximal interphalangeal (PIP), and metacarpophalangeal (MCP) joints. The thumb part consists of interphalangeal (IP), metacar-pophalangeal (MCP), and carpometacarpal (CMC) joints. Representative features of the HRI hand are as follows:

• Each finger is modular, so they can be combined in various forms.
• The robot finger has an under-actuated mechanism, the MCP joint is operated with one motor, and the PIP and DIP joints operate dependently.
• The wrist of the robot hand is based on ISO 9409–1-50–4-M6; therefore, it is compatible with robot arms of this specification.

The robot hand introduced in this paper is intended to be combined with a UR3 manipulator (Fig. 2c) and used for various applications. The dimensions of the HRI hand system are 84 mm × 61 mm × 235.5 mm, the dimensions of each finger are 13.16 mm × 13.2 mm × 82 mm, and the total weight is 570 g. These values are similar to the average adult male's hand and finger size. The detailed specifications are shown in Table 1.

**Table 1:** Specifications of the HRI hand.

Index	Specification
Weight of the HRI hand	570 g
Weight of the finger module	48 g
HRI hand configuration	5 fingers, 6 linear motors
Operating voltage	12 V
Degrees of freedom (DoF)	15
Communication range	<20 m
Size of the HRI hand	84 mm × 61 mm × 235.5 mm (W × L × H)
Size of the finger module	13.16 mm × 13.2 mm × 82 mm (W × L × H)
Microcontroller unit (MCU)	STM32F303 (ARM Cortex-M4)
Fingertip force (max.)	8.76 N
Finger speed (max.)	185.10°/s

**Fig. 3:**
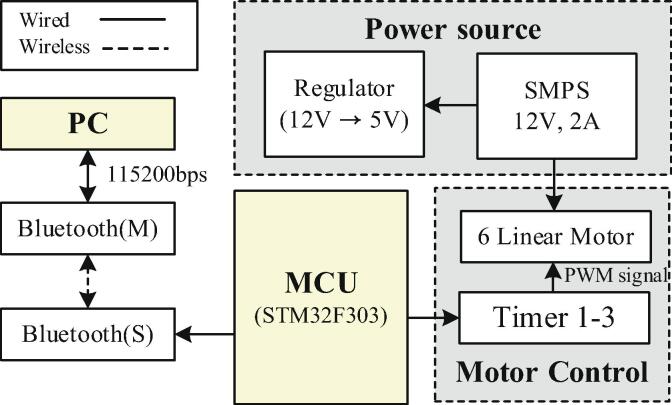
The architecture of the HRI hand system.

The control architecture is as follows and outlined in Fig. 3. The MCU uses the STM32F303 (32-bit processor, 64 MHz). The switching mode power supply (SMPS) is supplied with 12 V and 2 A of power, which is connected to a linear motor that operates the robot finger. To supply power to the MCU, a step-down regulator is used to convert from 12 V to 5 V. The PWM signal is sent to the motor at Timer 1–3 of the MCU and controlled. For communication with the Bluetooth module, UART 1 is set to 115,200 bps. At this time, the firmware upload uses UART 2 to prevent collision. The Bluetooth module consists of a master and a slave; the slave module connects with the MCU, and the master module connects with a PC to enable wireless communication. The data protocol for controlling the robot finger is as shown in Table 2.

**Table 2:** Data protocol for controlling HRI hand.

	Type	Size	Detail
Motor ID	Unsigned integer	8 bit	1 to 6
Motor PWM signal	Unsigned integer	16 bit	500 to 1000

**Fig. 4:**
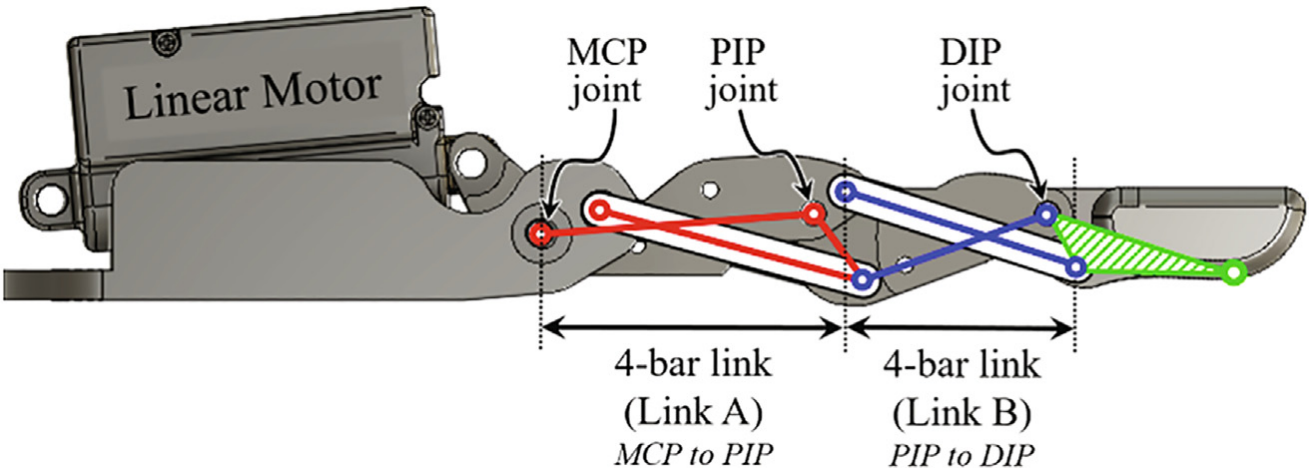
Design of the finger module.

As shown in Fig. 4, a finger module consists of the four links and three joints (MCP, PIP, and DIP joints). Since the finger module is an under-actuated system based on the two four-bar linkage mechanism, the MCP and PIP joints are connected to a four-bar link (Link A). Additionally, the PIP and DIP joints are connected to a four-bar link (Link B). The PIP and DIP joints operate dependently by the motor connected with the MCP joint.

**Fig. 5:**
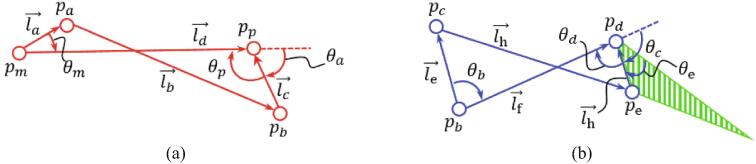
Kinematic diagram of the proposed finger module: (a) kinematic diagram of Link A, (b) kinematic diagram of Link B.

Fig. 5 shows the kinematic diagram of the finger module and $\vec{l_{d}}$ and $\vec{l_{f}}$ are fixed links; therefore, $\theta_{p}$ and $\theta_{d}$ are presented as equations (1)-(2). Additionally, we explain equations (1)-(2) in detail in the appendix.

(1) $$\theta_{p}=f\left(\theta_{a}\right)$$

(2) $$\theta_{d}=g\left(\theta_{c}\right)$$

**Fig. 6:**
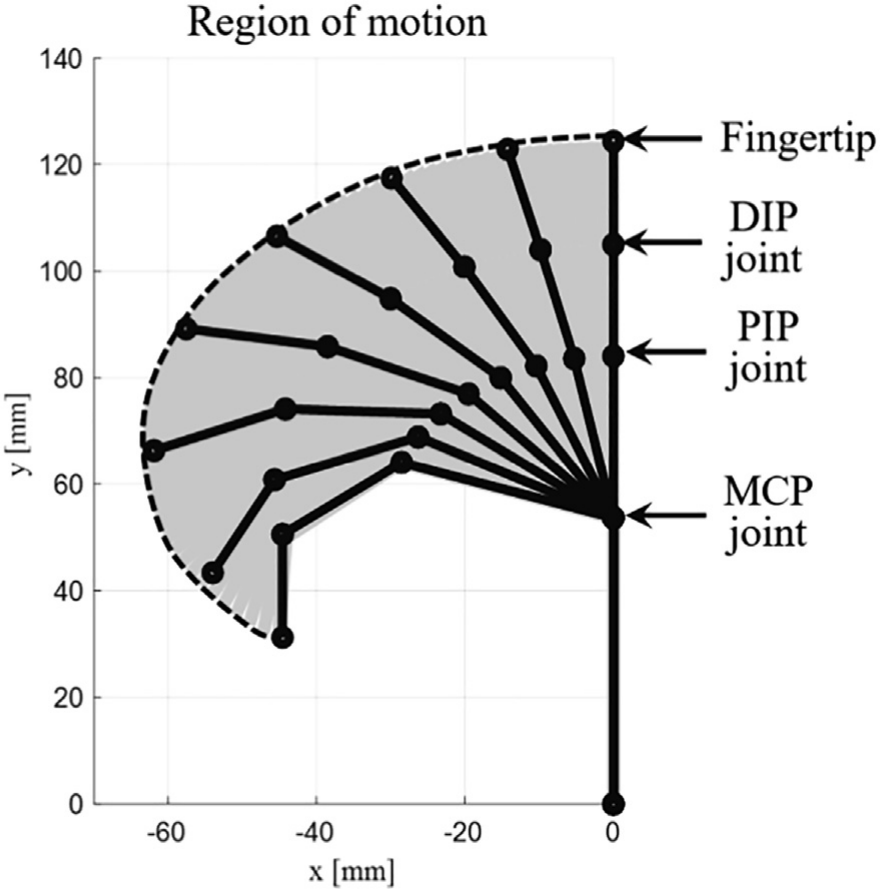
Region of motion about the finger module.

Fig. 6 shows the results of the position analysis of the finger module based on equations (1)-(2). The thick solid line is the position of the finger module, the thick solid circle is the position of the joints, the dashed line is the fingertip trajectory, and the gray region represents the region of motion about the finger module. Table 3 compares the characteristics of the HRI hand with that of commercial robot hands.

**Table 3:** Robot hand comparison.

Robot hand	Fingers	Actuator	Weight (kg)	Size (W × L × H mm)	Type
Schunk SDH Hand [23]	3	7	1.95	70 × 70 × 248.8	Gripper
Schunk SVH Hand [23]	5	9	1.3	90 × 90 × 242.5	Anthropomorphic
Barrett Hand [24]	3	4	1.2	110 × 335 × 119	Gripper
Robotiq (2-finger) [21]	2	1	1	206.9 × 35 × 209.8	Gripper
Robotiq (3-finger) [21]	3	2	2.3	155 × 111 × 204	Gripper
Shadow Dexterous [25]	5	20	4.3	84 × 135 × 448	Anthropomorphic
Allegro Hand [26]	4	16	1.5	139.5 × 40.8 × 247.7	Anthropomorphic
**HRI Hand**	**5**	**6**	**0.57**	**84**×**61**×**235.5**	**Anthropomorphic**

## 3 Design files

The hardware design files for the HRI hand system are summarized in Table 4 and the software components are summarized in Table 5.

**Table 4:** Design file summary for the HRI hand.

Design file name	File type	Open source license	Location of the file
palm_cover_2	STL, STEP, F3D	MIT License	https://osf.io/6kx4u/
F02_back_hand	STL, STEP, F3D	MIT License	https://osf.io/bv6a9/
F03_AL_palm	STL, STEP, F3D	MIT License	https://osf.io/p9q8y/
F03_Finger_base	STL, STEP, F3D	MIT License	https://osf.io/ekt82/
F03_MC_AL_Finger_01	STL, STEP, F3D	MIT License	https://osf.io/6sdt4/
F03_MC_AL_Finger_02	STL, STEP, F3D	MIT License	https://osf.io/9ghf4/
F03_PI_AL_Finger_01	STL, STEP, F3D	MIT License	https://osf.io/hfcta/
F03_PI_AL_Finger_02	STL, STEP, F3D	MIT License	https://osf.io/zcxb2/
F04_DP_AL_Finger	STL, STEP, F3D	MIT License	https://osf.io/ubpcx/
F03_pip_mcp link	STL, STEP, F3D	MIT License	https://osf.io/tyhve/
F03_dip_pip link	STL, STEP, F3D	MIT License	https://osf.io/sg8v9/
F03_Thumb_base	STL, STEP, F3D	MIT License	https://osf.io/t274y/
F03_Thumb_joint	STL, STEP, F3D	MIT License	https://osf.io/8mu76/
F02_palm_bottom	STL, STEP, F3D	MIT License	https://osf.io/dpe5h/
FR12_S102	STEP, F3D	MIT License	https://osf.io/8svwb/
ISO 9409-1-50-4-M6	STL, STEP, F3D	MIT License	https://osf.io/xwfbc/
motor_bracket	STEP, F3D	MIT License	https://osf.io/hb2yf/
pq-12_in	STEP, F3D	MIT License	https://osf.io/34zj8/
HRI_Hand_all_asm	STEP	MIT License	https://osf.io/bjzvt/

**Table 5:** Repositories for software components.

Software component	File type	Open source license	Location of the file
MCU firmware of the HRI hand	True studio and Cube MX	MIT License	OSF link: https://osf.io/vewp3/
			GitHub Repository: https://github.com/MrLacuqer/HRI-hand-firmware.git
Electronic schematic	Altium designer	MIT License	OSF link: https://osf.io/7q9sx/
			GitHub Repository: https://github.com/MrLacuqer/HRI-hand-firmware.git
ROS packages	Rviz launch, URDF xacro, python node	MIT License	OSF link: https://osf.io/vjrfh/
			GitHub Repository: https://github.com/MrLacuqer/HRI-Hand-ROS.git

### 3.1 Hardware files summary

• Four-finger part (index, middle, ring, little):
  The four fingers are configured by assembling ‘F03_Finger_base’, ‘F03_MC_AL_Finger_01, 02’, ‘F03_PI_AL_Finger_01, 02’, and ‘F04_DP_AL_Finger’.
  ‘F03_pip_mcp link’ connects the PIP joint and the MCP joint.
  ‘F03_dip_pip link’ connects the DIP joint with the PIP joint.
• Thumb part:
  The thumb part is configured by assembling ‘F03_Thumb_base', ‘F03_Thumb_joint’, and ‘F03_MC_AL_Finger_01, 02’.
  ‘F03_pip_mcp link’ connects the IP joint and the MCP joint.
• The others:
  The palm of the HRI hand is configured by assembling ‘F03_AL_palm’ and ‘palm_cover_2’.
  The wrist part is configured by assembling ‘F02_palm_bottom', ‘FR12_S102’, and ‘ISO 9409–1-50–4-M6’.

### 3.2 Software files summary

• MCU firmware of the HRI hand: the MCU firmware uses HAL driver API from STMicroelectronics and the complier using TrueSTUDIO for STM32.
• Electronic schematic: using Altium Designer, “hri_hand_schematic.SchDoc” is the electronic circuit, and “hri_hand_v2_1.PcbDoc” is the PCB layout.
• ROS packages: include the URDF xacro file of the HRI hand, Rviz visualization launch files, and python node for controlling the HRI hand.

## 4 Bill of materials

The bill of materials for this project is summarized in Table 6.

**Table 6:** The Bill of materials for the HRI hand system.

Designator	Component	Number	Cost per unit	Total cost	Source of materials	Material type
Micro-controller unit	NUCLEO-F303K8	1	$10.33	$10.33	Mouser electronics	Others
Designator	Component	Number	Cost per unit	Total cost	Source of materials	Material type
Linear motor	PQ12 Linear Actuator 20 mm, 100:1, 12 V, RC Control	6	$70	$420	Robotshop	Others
Bluetooth module	HC-06	2	$3.48	$6.96	eBay	Others
Step-down voltage regulator	Pololu S10V4F5	1	$4.49	$4.49	Pololu	Others
Wrist bracket	Robotis Co. braket FR12-S102K Set	1	$15.90	$15.90	Robotis	Aluminum
USB-to-serial adapter	Pololu: USB-to-Serial Adapter	1	$14.95	$14.95	Pololu	Others
SUS304 processing cost	Design file name: F03_MC_AL_FINGER_01, 10ea. F03_MC_AL_FINGER_02, 8ea. F03_PI_AL_FINGER_01, 8ea. F03_PI_AL_FINGER_02, 8ea. F03_AL_PALM, 1ea.	1	$440.73	$440.73	Robotnmore Co. (Korean company)	SUS304

A list of all of the components used in this project can be found in the BOM spreadsheet: https://osf.io/2zybw/.

## 5 Build instructions

### 5.1 HRI hand assembly

**Fig. 7:**
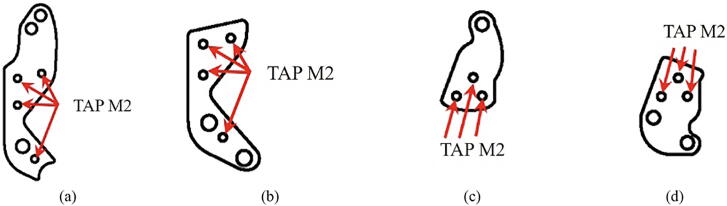
The point of tap processing: (a) F03_MC_AL_Finger_01, (b) F03_MC_AL_Finger_02, (c) F03_PI_AL_Finger_01, and (d) F03_PI_AL_Finger_02.

**Fig. 8:**
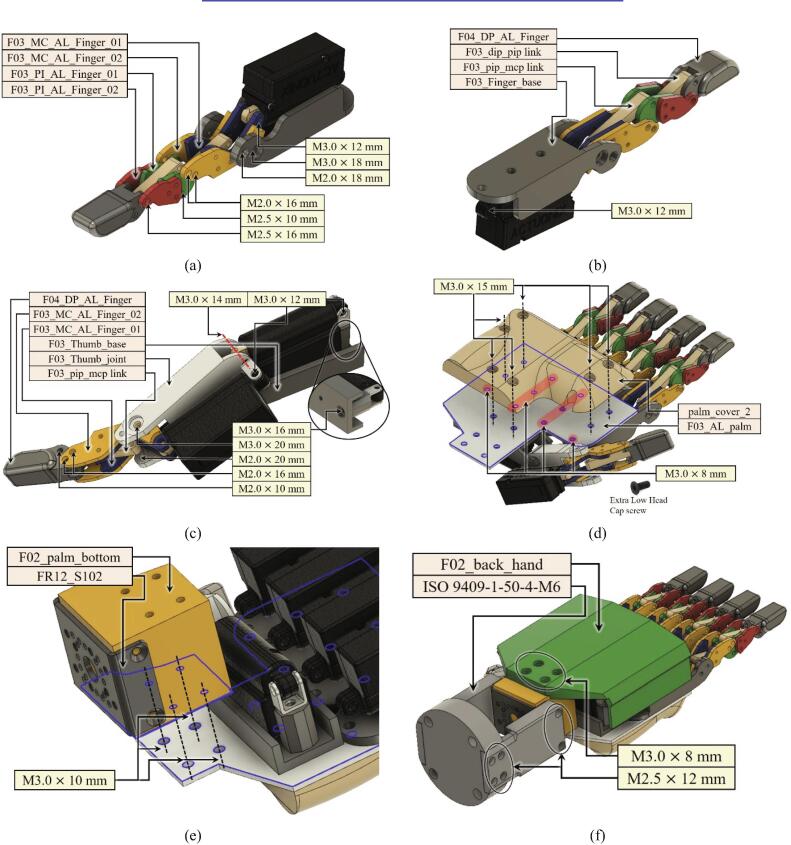
Assembly procedures for the HRI hand: (a) front of the finger, (b) back of the finger, (c) thumb part, (d) palm and palm cover, (e) wrist part, (f) back of the HRI hand cover and ISO 9409-1-50-4-M6 part.

The total assembly process of the HRI hand is carried out in the order outlined in Fig. 8a-f. Several additional processing steps are required before the assembly of the HRI hand begins. First, for the components of the robot finger, ‘F03_MC_AL_Finger_01, 02’ and ‘F03_PI_AL_Finger_01, 02’, tap processing is required as shown in Fig. 7, and it is combined with headless-bolt M2.0 × 4 mm. 'F02_palm_botom' in Fig. 8e also needs tap processing in all holes. M3.0 × 8 mm bolts used in Fig. 8d are recommended for use with extra low head cap screws. As shown in Fig. 8a, the robot finger is modular. It can be composed of various types of end-effectors based on the purpose of the user as well as the ease of maintenance.

### 5.2 Configuration of electronic schematic

**Fig. 9:**
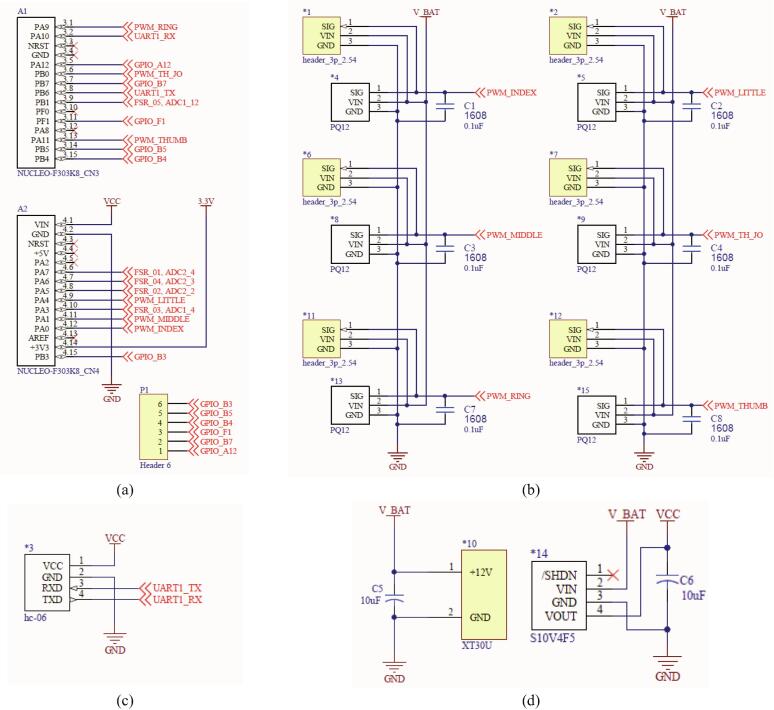
The key point electronic schematic of the HRI hand system: (a) MCU part, (b) six linear motor part, (c) Bluetooth module part, (d) power source part.

**Fig. 10:**
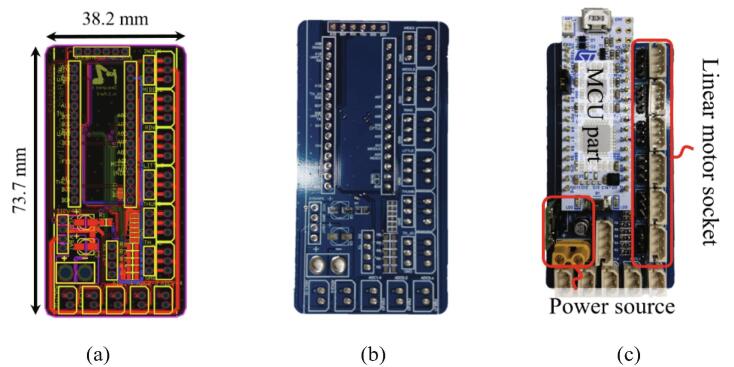
The PCB layout: (a) PCB layout in the ECAD, (b) PCB board, (c) the PCB assembled with each component.

The control board of the HRI hand controls six linear motors and is controlled based on data as shown in Table 2 through the Bluetooth module. The electronic schematic of this control board is as shown in Fig. 9, and the layout of the PCB board is shown in Fig. 10. All the schematics for the HRI hand system are available at the following open-source websites:

• OSF link: https://osf.io/wudtf/
• GitHub repository: https://github.com/MrLacuqer/HRI-hand-firmware.git

## 6 Operation instructions

In this section, we discuss how to operate the hardware. At the time of writing, snapshots of the firmware, configuration files, and software have been stored in the project’s repository on the Open Science Foundation’s website. These snapshots are the versions of the software referred to in this article. More recent versions of this software may be found in the GitHub repositories listed in Table 3.

### 6.1 Operation procedure

**Fig. 11:**
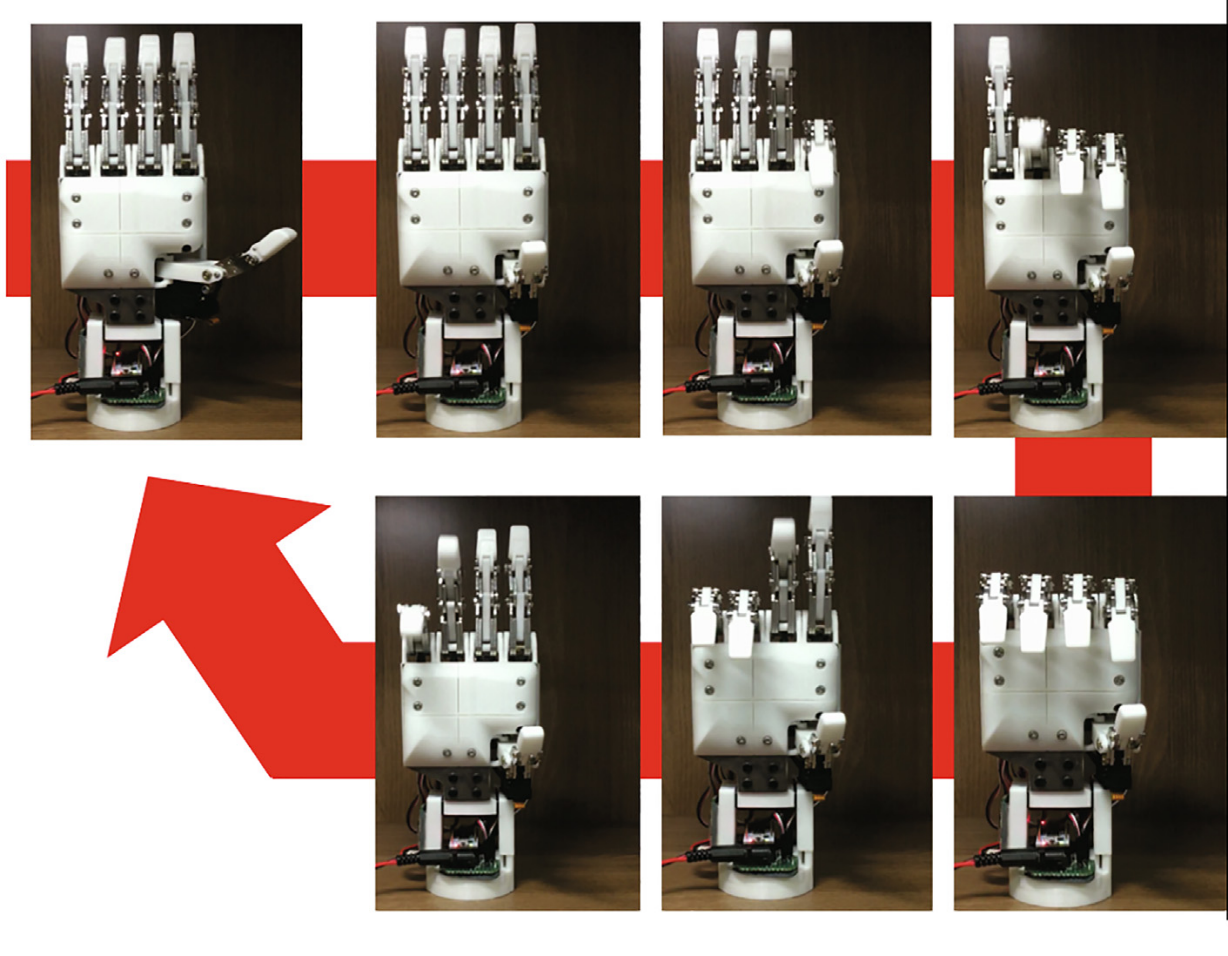
The initializing process after successful uploading on the MCU.

• Download the MCU firmware of the HRI hand in the repositories listed in Table 3.
• Connect the NUCLEO-F303K8 to the PC and firmware uploading to the NUCLEO-F303K8.
• If successful in uploading the firmware to the NUCLEO-F303K8, each finger of the HRI hand will complete a bending motion one by one (Fig. 11).

### 6.2 Robot Operating system (ROS) package procedure

The HRI hand system is interoperable with the ROS. For this, the Unified Robot Description Format (URDF) and the visualization package are configured. The URDF is the description of a robot consisting of a set of link (part) elements, and a set of joint elements connecting the links together, which is an XML format [27]. The visualization package consists of the “robot state publisher”, “joint state publisher”, and “rviz”. The robot state publisher is publishing the transformation of the robot based on URDF file, and the joint state publisher is publishing the joint position of the robot [28], [29]. The rviz is a ROS graphical interface that allows the user to visualize a lot of information [30], this package, visualizing the robot state and joint state. There is also a python node that can control the HRI hand, which can be implemented through the following process. The python node executes the motor control signal, robot state, and joint state of each finger.

• Install ‘Ubuntu 16.04’ and ‘ROS Kinetic’ on a computer.
• Connect the USB-to-serial adapter to the PC and type the following command:

**Table:** 

$ cd ∼/catkin_ws/src && git clone https://github.com/MrLacuqer/HRI-Hand-ROS.git
$ cd ∼/catkin_ws && catkin_make
$ rospack profile && rosstack profile
$ roslaunch hri_hand_control hri_hand_control.launch
$ rosrun hri_hand_control hri_joint_state_pub.py

## 7 Validation and characterization

**Fig. 12:**
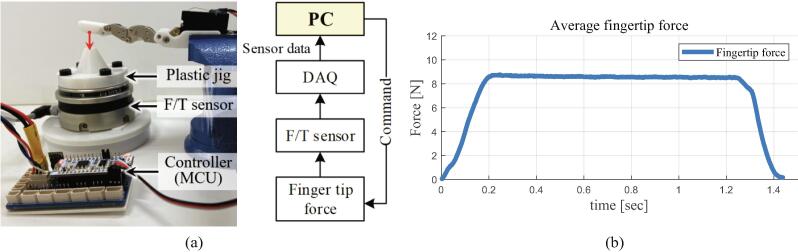
Fingertip force experiment setup and results: (a) experimental environment, (b) results of the average fingertip force plot.

The proposed HRI hand system is developed with a five-finger structure, but each finger is modularized, so it can be developed with end-effector with various shapes depending on the shape of the palm. Therefore, the grasping force limit of the end-effector is different according to the finger module combination. The fingertip force is measured by an F/T (force/torque) sensor (HEX-70-XE-200 N, Optoforce Co., Denmark), as shown in Fig. 12a. The sensor data is transmitted through the DAQ (data acquisition) device to the PC when the finger module presses the plastic jig. The plastic jig is mounted to distribute the pressure to the F/T sensor equally. The finger module generates 8.76 N at the peak, as shown in Fig. 12b.

**Fig. 13:**
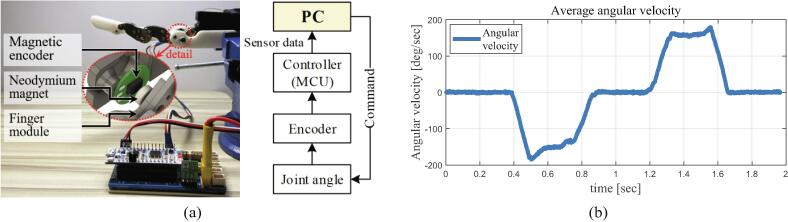
Angular velocity experiment setup and results: (a) experimental environment, (b) results of the average angular velocity plot.

To verify the maximum flexion/extension speed, we developed an experimental environment, as shown in Fig. 13a. The angle of the MCP joint is measured from the magnetic encoder (EzEncoder, i2A Systems Co., South Korea), and the measured angle is differentiated according to time to calculate the angular velocity. As a result, as shown in Fig. 13b, the maximum velocity of the bending motion is 185.10°/s, and the maximum velocity of the extension motion is 179.50°/s. All experiments are performed ten times.

**Fig. 14:**
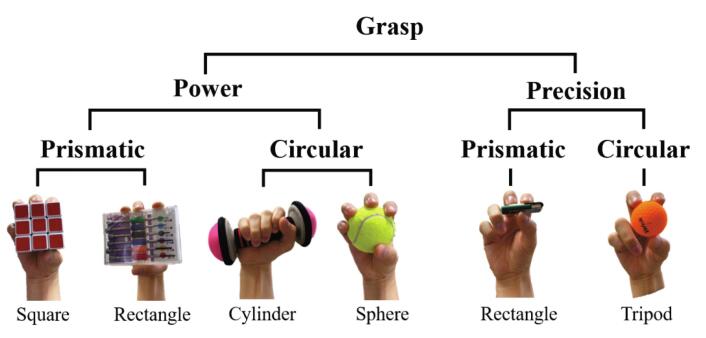
The grasp taxonomy derived from [31], [32], [33].

**Fig. 15:**
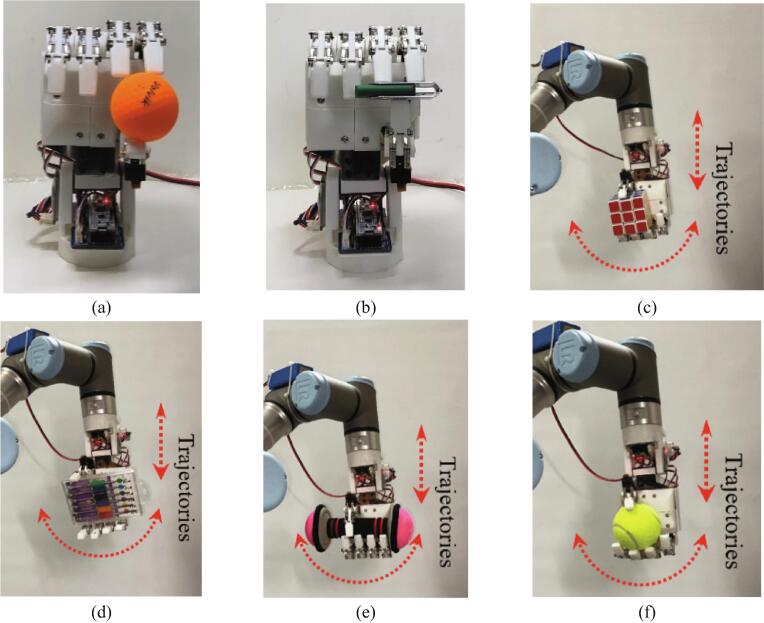
Illustration of the grasp performance: precision grasp (a) tripod type and (b) rectangle type, and power grasp (c) square type, (d) rectangle type, (e) cylinder type, and (f) sphere type.

To verify the object grasping of the HRI hand, as shown in Fig. 14, we have determined the six grasp types following [31], [32], [33]. The detail size and weight of the grasp objects shown in Table 7. The precision grasp is an experiment to verify dexterity and sensitivity (Fig. 15a-b). In contrast, the power grasp is important to maintain robust grasping despite the operation of the manipulator. Therefore, as shown in Fig. 15c-f, power grasping is verified through up-down and swinging motions after the HRI hand is mounted on the manipulator. All grasping experiments were successful.

**Table 7:** The size and weight of the grasp objects.

Grasp type	Power grasp				Precision grasp	
Object type	Square	Rectangle	Cylinder	Sphere	Rectangle	Tripod
Size [mm]	57 × 57 × 57 (W × L × H)	85 × 123 × 20 (W × L × H)	∅32	∅63.8	20 × 54.8 × 10 (W × L × H)	∅42.5
Weight [g]	59.5	136	1011	56.5	14	45.5

All experimental videos are available at the following links:

• **The HRI hand with ROS rviz package test:**
  https://youtu.be/vD6ZCrParco
• **The HRI hand grasping test:**
  https://youtu.be/c5Ry3tl9FVw

**Fig. 16:**
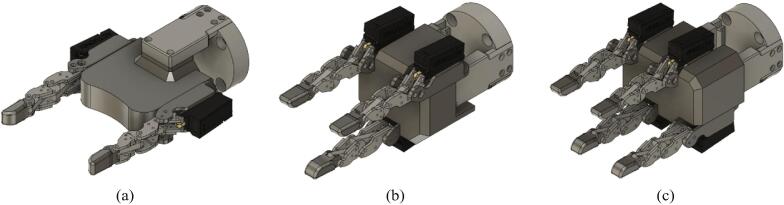
Various end-effectors: (a) two fingered end-effector, (b) three fingered end-effector, (c) four fingered end-effector.

The robot finger is modularized; researchers can use it as an end-effector of a desired shape according to the design of the palm as shown as Fig. 16.

## 8 Conclusions

In this paper, we presented an open-source anthropomorphic robot hand system called HRI hand. Our robot hand system is developed with a focus on the end-effector role of the collaborative robot manipulator. Since the proposed robot hand imitated the human hand, the four fingers, excluding the thumb, consist of DIP, PIP, and MCP joints. The HRI hand is a research platform that can be built at a lower price (approximately $500, using only 3D printing) than a commercial end-effector. Moreover, it is designed as a two four-bar linkage for the under-actuated mechanism and provides pre-shaping motion similar to the human hand prior to touching an object. The thumb part consists of IP, MCP, and CMC joints, and operates MCP and CMC joints with two motors. The motor is controlled based on the control signal received by the micro-controller unit (MCU) via Bluetooth communication. A URDF, python node, and rviz package is also provided to support the Robot Operating System (ROS). All hardware CAD design files and software source codes have been released and can be easily assembled and modified.

The system proposed in this paper is developed with a five-finger structure, but each finger is modularized, so it can be developed with end-effectors of various shapes depending on the shape of the palm. For example, it is possible to construct various types of end-effectors depending on the researcher's purpose, such as two-fingered grippers with two fingers or three-fingered grippers with three fingers. For those interested in implementing a variety of robot applications using the proposed system, we would strongly encourage contacting the corresponding author to discuss potential collaboration.

## Declaration of Competing Interest

The authors declare that they have no known competing financial interests or personal relationships that could have appeared to influence the work reported in this paper.

## Appendix A

In Fig. 6, $\theta_{p}$ and $\theta_{d}$ are calculated as follows:

(3) $$\theta_{p}=\pi -\theta_{a}$$

(4) $$\theta_{d}=\left(\pi -\theta_{c}\right)+\theta_{e}$$

The ratio of Link A and Link B are presented in equation (5).

(5-1) $$k_{1}=\frac{l_{d}}{l_{a}}$$

(5-2) $$k_{2}=\frac{l_{d}}{l_{c}}$$

(5-3) $$k_{3}=\frac{l_{a}^{2}-l_{b}^{2}+l_{c}^{2}+l_{d}^{2}}{2l_{a}l_{c}}$$

(5-4) $$k_{4}=\frac{l_{h}}{l_{e}}$$

(5-5) $$k_{5}=\frac{l_{h}}{l_{g}}$$

(5-6) $$k_{6}=\frac{l_{e}^{2}-l_{f}^{2}+l_{g}^{2}+l_{h}^{2}}{2l_{e}l_{g}}$$

As a result, $\theta_{a}$ and $\theta_{c}$ are presented in equation (6).

(6-1) $$\begin{array}{c} \theta_{a} \end{array}=\begin{array}{c} 2\tan^{-1}\left(\frac{-\beta +\sqrt{\beta^{2}-4\alpha \gamma }}{2\alpha }\right) \end{array}$$

(6-2) $$\begin{array}{c} \theta_{c} \end{array}=\begin{array}{c} 2\tan^{-1}\left(\frac{-\varepsilon +\sqrt{\varepsilon^{2}-4\delta \zeta }}{2\delta }\right) \end{array}$$

where α,β,γ,δ,ε,ζ are as follows:

$$\begin{array}{c} \alpha \end{array}=\cos\theta_{m}-k_{1}-k_{2}\cos\theta_{m}+k_{3}$$

$$\beta =-2\sin\theta_{m}$$

$$\gamma =k_{1}-\left(k_{2}+1\right)\cos\theta_{m}+k_{3}$$

$$\begin{array}{c} \delta \end{array}=\cos\theta_{b}-k_{3}-k_{4}\cos\theta_{b}+k_{5}$$

$$\varepsilon =-2\sin\theta_{b}$$

$$\zeta =k_{4}-\left(k_{5}+1\right)\cos\theta_{b}+k_{6}$$
