# Supplementary figures and images for: An open-source anthropomorphic robot hand system: HRI hand
Source: HardwareX. 2020 Feb 24;7:e00100. doi: 10.1016/j.ohx.2020.e00100 (PMC9041201; doi:10.1016/j.ohx.2020.e00100)

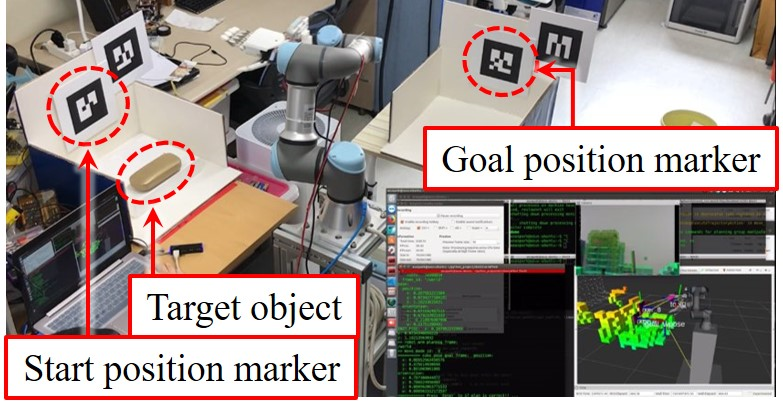

Supplement: Supplementary data 3 [file mmc3.zip › images/fig10b.png]

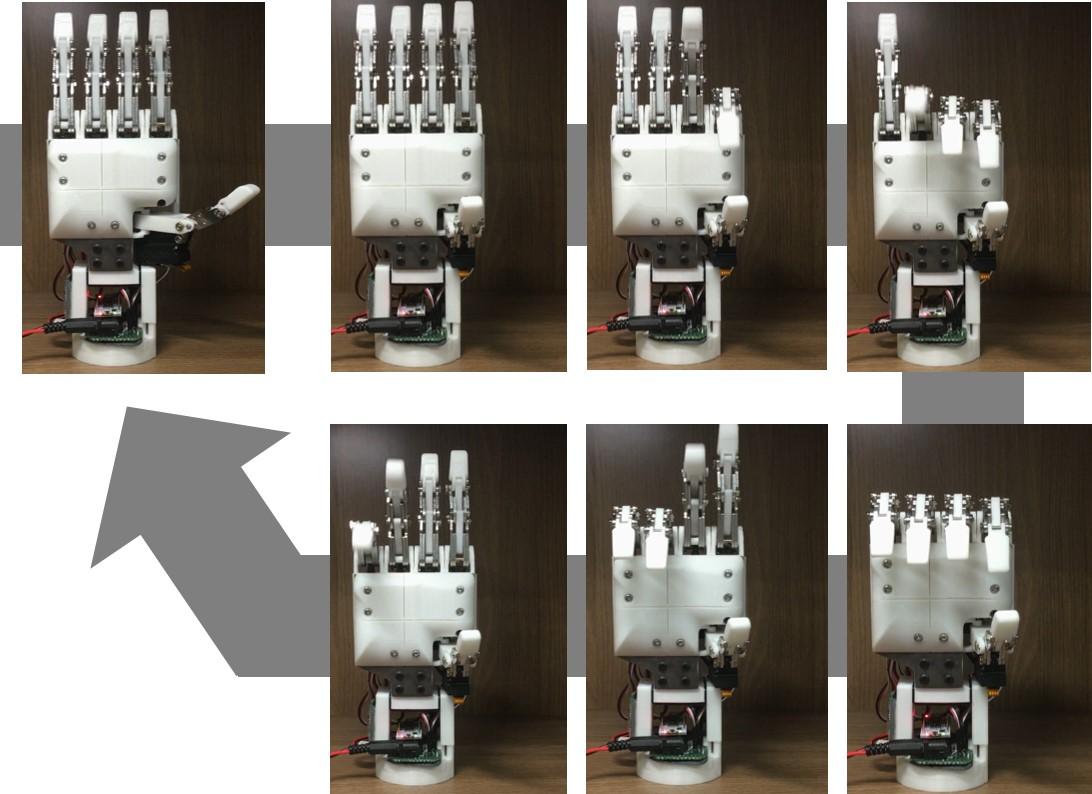

Supplement: Supplementary data 3 [file mmc3.zip › images/fig8.png]

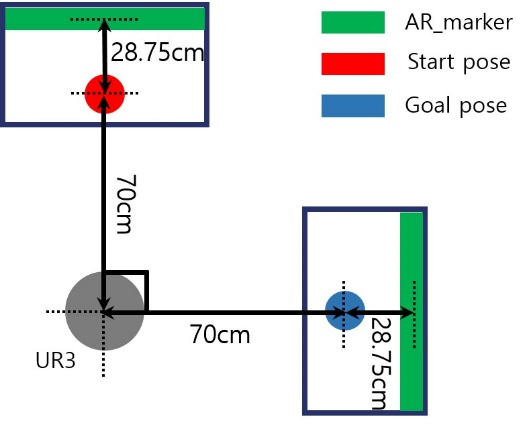

Supplement: Supplementary data 3 [file mmc3.zip › images/fig10a.png]

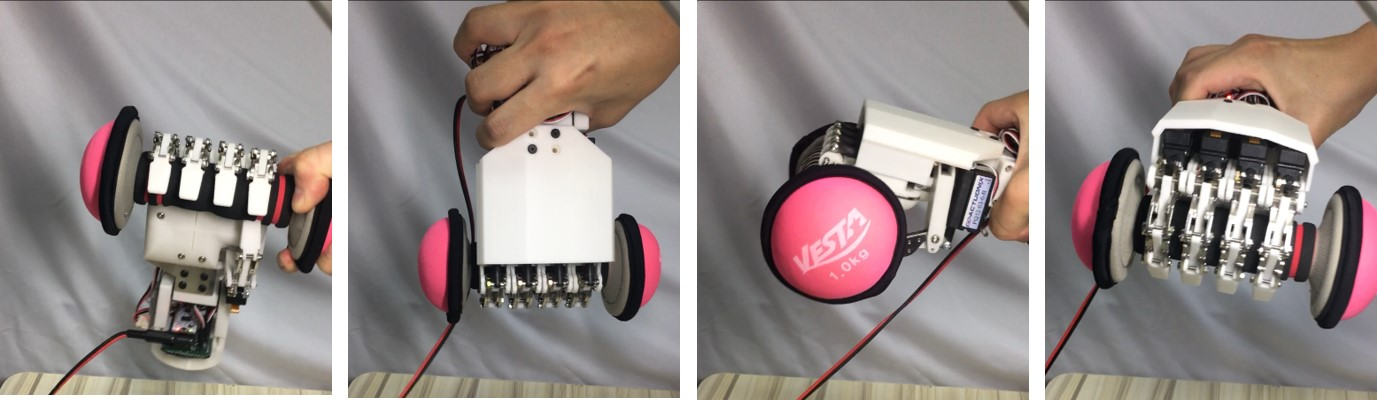

Supplement: Supplementary data 3 [file mmc3.zip › images/fig9.png]

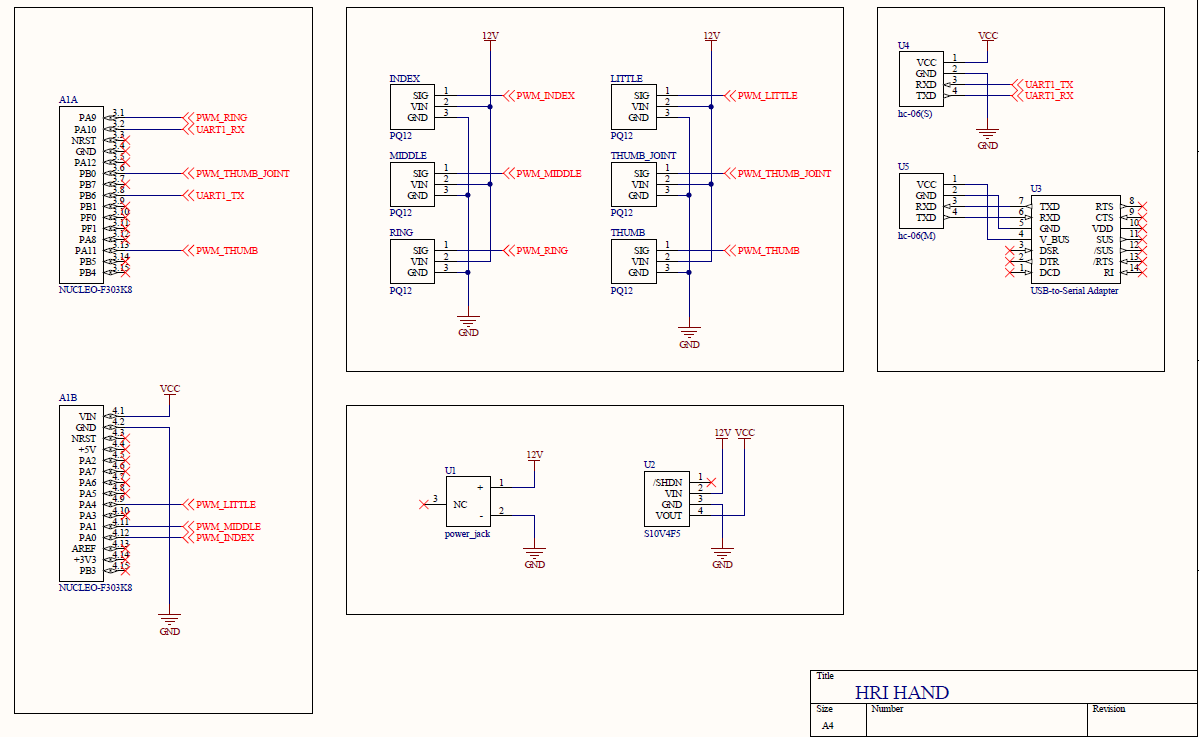

Supplement: Supplementary data 3 [file mmc3.zip › images/fig7.png]

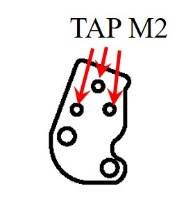

Supplement: Supplementary data 3 [file mmc3.zip › images/fig6d.png]

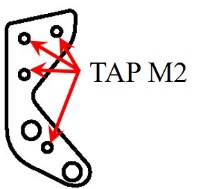

Supplement: Supplementary data 3 [file mmc3.zip › images/fig6b.png]

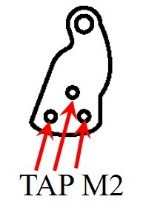

Supplement: Supplementary data 3 [file mmc3.zip › images/fig6c.png]

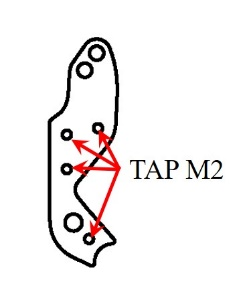

Supplement: Supplementary data 3 [file mmc3.zip › images/fig6a.png]

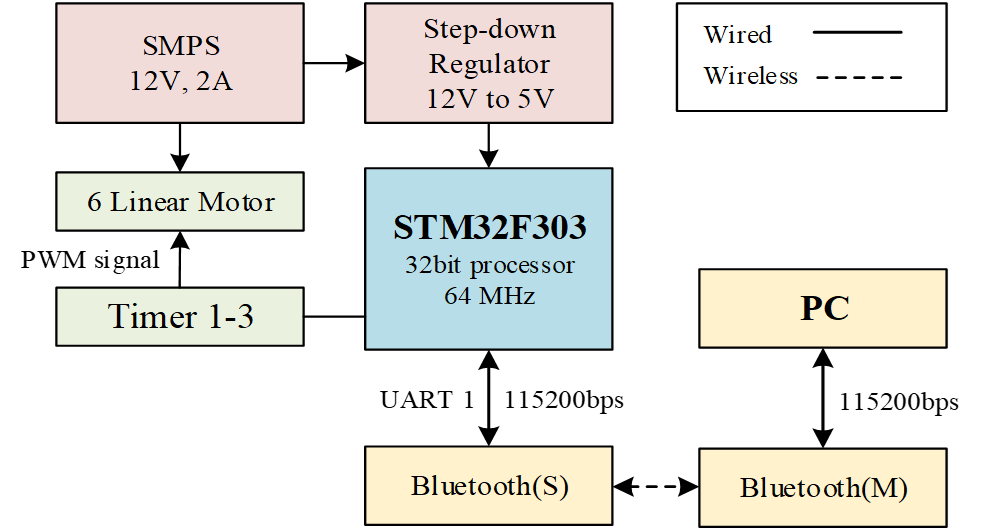

Supplement: Supplementary data 3 [file mmc3.zip › images/fig4.tif]

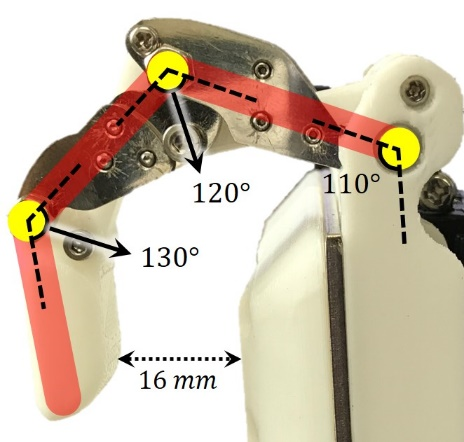

Supplement: Supplementary data 3 [file mmc3.zip › images/fig3.png]

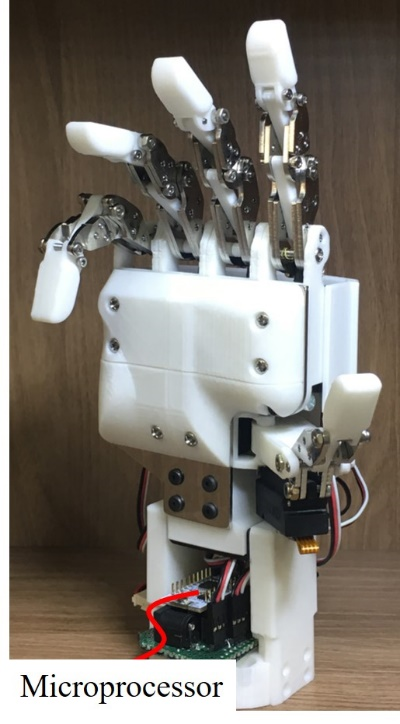

Supplement: Supplementary data 3 [file mmc3.zip › images/fig2_b.png]

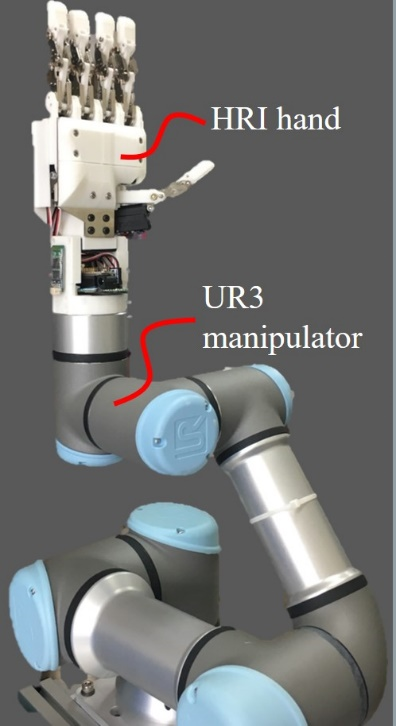

Supplement: Supplementary data 3 [file mmc3.zip › images/fig2_c.png]

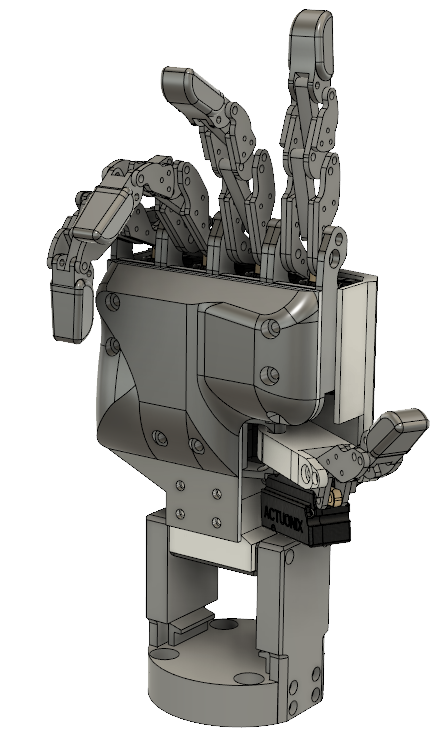

Supplement: Supplementary data 3 [file mmc3.zip › images/fig2_a.png]

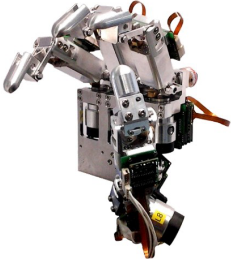

Supplement: Supplementary data 3 [file mmc3.zip › images/fig1b__3.png]

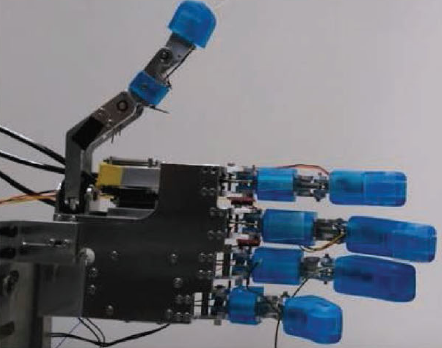

Supplement: Supplementary data 3 [file mmc3.zip › images/fig1b__4.png]

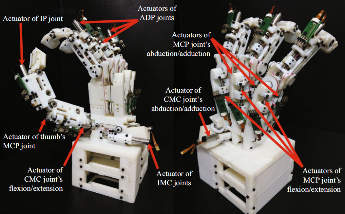

Supplement: Supplementary data 3 [file mmc3.zip › images/fig1b__2.png]

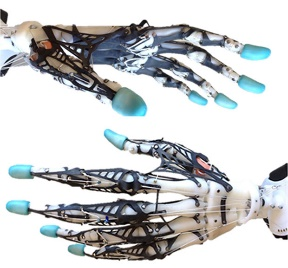

Supplement: Supplementary data 3 [file mmc3.zip › images/fig1b__1.png]

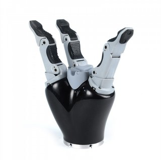

Supplement: Supplementary data 3 [file mmc3.zip › images/fig1a__4.png]

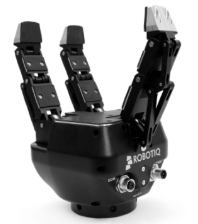

Supplement: Supplementary data 3 [file mmc3.zip › images/fig1a__3.png]

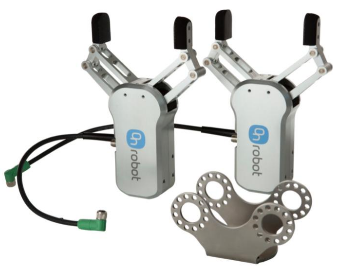

Supplement: Supplementary data 3 [file mmc3.zip › images/fig1a__2.png]

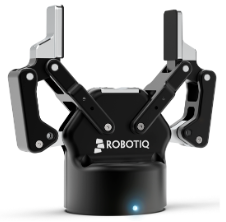

Supplement: Supplementary data 3 [file mmc3.zip › images/fig1a__1.png]

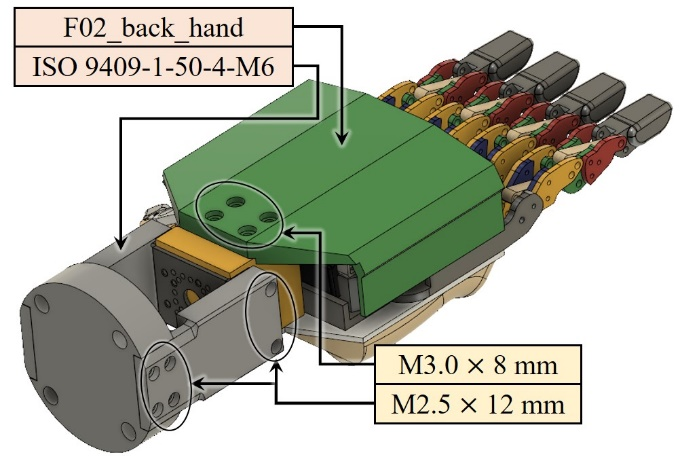

Supplement: Supplementary data 3 [file mmc3.zip › images/fig.5f.png]

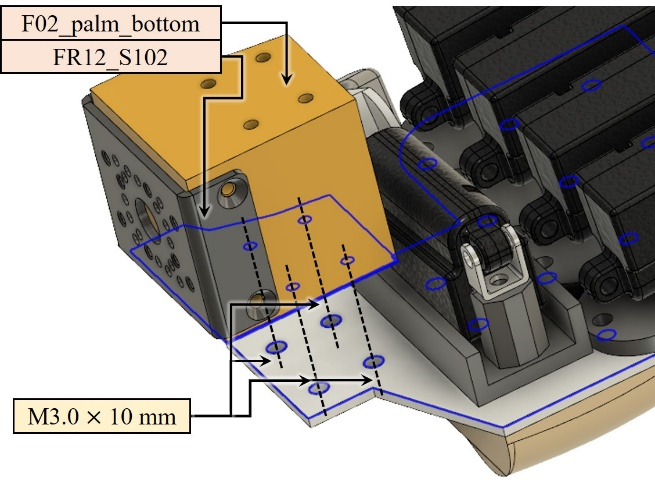

Supplement: Supplementary data 3 [file mmc3.zip › images/fig.5e.png]

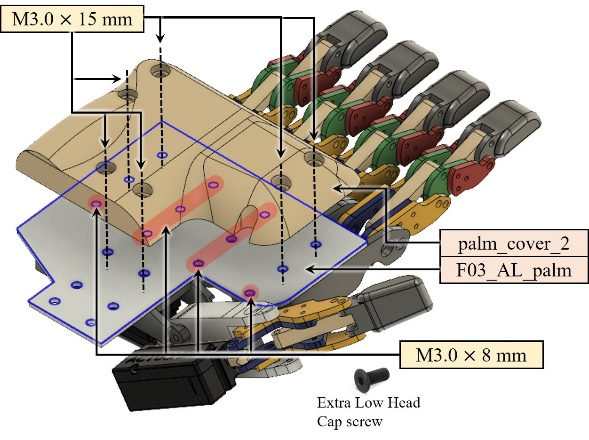

Supplement: Supplementary data 3 [file mmc3.zip › images/fig.5d.png]

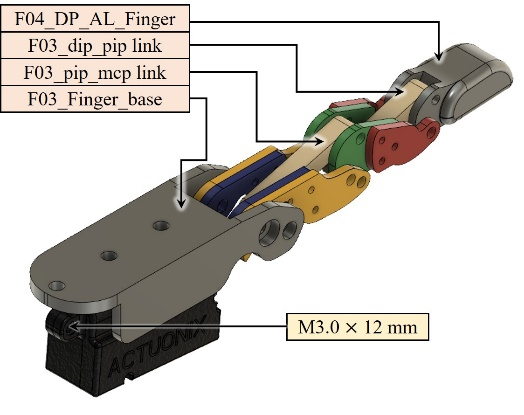

Supplement: Supplementary data 3 [file mmc3.zip › images/fig.5b.png]

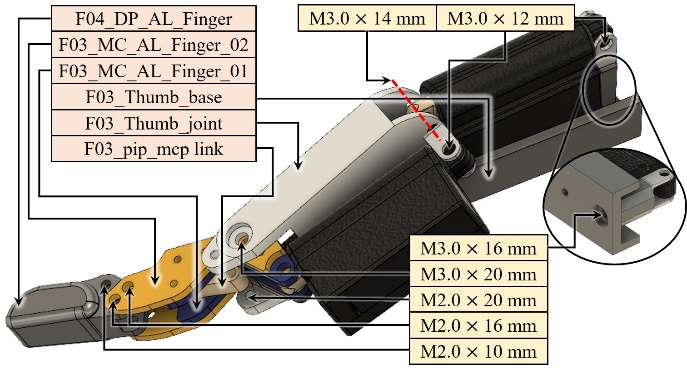

Supplement: Supplementary data 3 [file mmc3.zip › images/fig.5c.png]

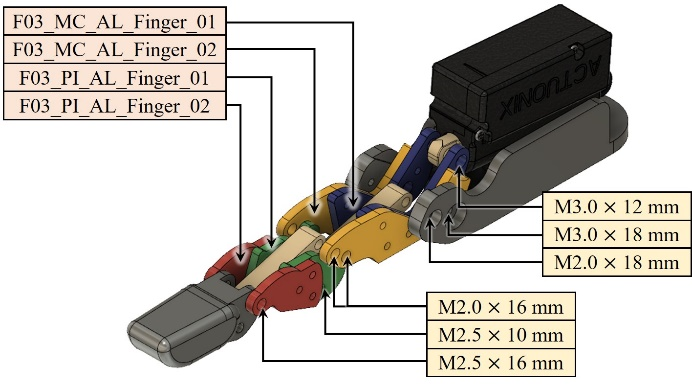

Supplement: Supplementary data 3 [file mmc3.zip › images/fig.5a.png]
